# Interaction Between Daidzein and Hesperetin on Antispasmodic Action in Isolated Sensitized and Non-sensitized Guinea-Pig Tracheas

**DOI:** 10.3389/fphar.2016.00075

**Published:** 2016-03-29

**Authors:** Chung-Hung Shih, Tsu-Ya Chang, Wun-Chang Ko

**Affiliations:** ^1^Department of Internal Medicine, Taipei Medical University HospitalTaipei, Taiwan; ^2^Department of Pharmacology, College of Medicine, Taipei Medical UniversityTaipei, Taiwan

**Keywords:** antagonism, daidzein, Guinea-pig trachea, hesperetin, isobole, synergism

## Abstract

In traditional Chinese medicine (TCM), a combination of kudzu and Chen-Pi is frequently prescribed for relieving colds, fever, bronchitis, and cough. It contains daidzein and hesperetin, selective inhibitors of family 3 (PDE3), and 4 (PDE4) of phosphodiesterases (PDEs), respectively. In passively sensitized human airways, allergen-induced contraction was reported to be inhibited only by the simultaneous inhibition of PDE3 and PDE4, but not by single inhibition of either isozyme. Therefore, we are interested in investigating the interaction between daidzein and hesperetin on their antispasmodic effects in the isolated sensitized and non-sensitized guinea-pig tracheas, to clarify the difference between these two tissues, because effects of TCM prescription on patients with or without allergic asthma are often different. Guinea-pigs were sensitized by subcutaneous injection of ovalbumin (OVA) into legs. After sensitization, the baseline and cumulative OVA-induced contractions of the sensitized trachea were isometrically recorded on a polygraph. In the same way, the histamine (30 μM)-induced tonic contraction of non-sensitized guinea-pig trachea was recorded. The isobole method was used to analyze the antagonism and synergism between daidzein and hesperetin. The isoboles showed antagonism between daidzein and hesperetin on baseline relaxant effect and OVA (100 μg/ml)-induced contraction in the sensitized guinea-pig trachea. In contrast, the isobole showed synergism between daidzein and hesperetin on the relaxant effect of histamine-induced tonic contraction in non-sensitized guinea-pig trachea. These results suggest that the combination of kudzu and Chen-Pi for relieving colds, fever, bronchitis and cough is effective in patients without, but might show little effect in patients with allergic asthma.

## Introduction

Daidzein (7,4′-dihydroxyisoflavone) is about 1% present in the tuber of kudzu (*Pueraria pseudo-hirsuta* Tang et Wang, *Leguminosae*), known as Radix *Puerariae*. In TCM, kudzu is used against colds, fever, influenza, diarrhea, urticaria, tonsillitis, headache, and spasm (Yen, 1970). The alcoholic extract of kudzu or daidzein has been proved to have antispasmodic action against acetylcholine-induced contraction in isolated mouse intestine. The antispasmodic action is non-competitive and papaverine-like (Yen, 1970). Thus it may be a result from their selective inhibition of family 3 (Ko et al., 2004) of PDEs which consists of 11 families reported up today, and by which adenosine (cAMP) or guanosine cGMP is hydrolyzed to form inactive AMP or GMP. After inhibition of PDEs, cAMP or cGMP level is enhanced and subsequently activates cAMP- or cGMP-dependent protein kinase which may phosphorylate and inhibit myosin light-chain kinase, thus inhibiting contractions (Westfall et al., 1998). Hesperetin (5,7,3′-trihydroxy-4′-methoxyflavanone) is about 1.5% present in the fruit peel of *Citrus aurantium* L. (*Rutaceae*), well-known as “Chen-Pi” in TCM, which is used as an expectorant (Yen, 1971). Men with higher hesperetin intake have lower mortality from lung cancer, and lower incidences of asthma (Knekt et al., 2002). Allergic asthma is a chronic respiratory disease characterized by airway hyperresponsiveness (AHR), mucus hypersecretion, bronchial inflammation, and elevated immunoglobulin (Ig) E levels. T helper type-2 (Th2) cells, together with other inflammatory cells such as eosinophils, B cells, and mast cells are thought to play critical roles in the initiation, development, and chronicity of this disease (Busse and Lemanske, 2001). Hesperetin was reported to selectively inhibit PDE4 activity (Ko et al., 2004), and to have a suppressive effect on OVA-induced AHR (Shih et al., 2012). In TCM, a combination of kudzu and Chen-Pi is frequently prescribed for relieving colds, fever, bronchitis, and cough (Yen, 1971).

PDE3 and PDE4 isozymes are cGMP inhibited and cAMP specific, respectively. They were identified in the guinea pig airway (Silver et al., 1988). In passively sensitized human airways, allergen-induced contraction was inhibited only by the simultaneous inhibition of PDE3 and PDE4, but not by a single inhibition of either isozyme (Schmidt et al., 2000). Therefore, we were interested in investigating the interaction between daidzein and hesperetin in combination on their antispasmodic effects in the isolated sensitized and non-sensitized guinea-pig tracheas, to clarify the difference between these two tissues, because an effect of prescriptions in TCM on patients with or without allergic asthma is often different.

## Materials and Methods

### Reagents and Animals

Daidzein (mol. wt., 254.24; purity > 98%), hesperetin (mol. wt., 302.28; purity > 95%), aminophylline, histamine, indomethacin, nifedipine (purity > 98%) and OVA were purchased from Sigma–Aldrich. Freund’s complete adjuvant (*Mycobacterium butyricum*) was purchased from Pierce Biotechnology.

Male Hartley guinea pigs (500∼600 g) were purchased from the Animal Center of the Ministry of Science and Technology (Taipei, Taiwan), and housed in ordinary cages at 22 ± 1°C with a humidity of 50%∼60% under a constant 12/12-h light/dark cycle and provided with food and water *ad libitum*. Under a protocol (LAC-94-0091) approved by the Animal Care and Use Committee of Taipei Medical University on December 22, 2005, the guinea-pig tracheas were dissected under anesthesia (pentobarbital 50 mg/kg, intraperitoneal injection).

### Sensitized and Non-sensitized Tracheas

Guinea-pigs were subcutaneously injected a mixture of 10% OVA (w/v) and Freund’s complete adjuvant (1:1) into both sides of legs (0.7 ml for each side) on day 1 and 4. On day 25, these sensitized guinea-pigs were sacrificed to study their tracheal relaxations (Underwood et al., 1994). In the following experiments, the tension changes of guinea-pig tracheal segment were isometrically recorded (Ko et al., 2000). The sensitized tissues were contracted with 60 mM KCl and then washout three times with Krebs solution followed. The composition of Krebs solution was (mM): NaCl 118, KCl 4.7, MgSO_4_ 1.2, KH_2_PO_4_ 1.2, CaCl_2_ 2.5, NaHCO_3_ 25, and dextrose 10.1. Indomethacin (3 μM) was added to the Krebs solution to prevent the synthesis of prostaglandins throughout the experiment. The sensitized tissues were incubated with hesperetin, daidzein, combination (daidzein plus hesperetin 10, 30, and 100 μM each, daidzein 20 μM plus hesperetin 50, 75, and 100 μM, and daidzein 30 μM plus hesperetin 30, 60, and 100 μM) or its vehicle (control) for 15 min, and then cumulatively added OVA (0.01, 0.1, 1, 10, and 100 μg/ml). The effects of hesperetin, daidzein, combination, or its vehicle on the baseline tension and OVA-induced contractions were expressed as a percentage of 60 mM KCl-induced contraction (100%). In the dark, nifedipine (1 μM) was used as a reference drug. After the non-sensitized tissues were precontracted with histamine (30 μM), daidzein, hesperetin, combination (daidzein plus hesperetin 50, 75, and 100 μM each, daidzein 20 μM plus hesperetin 100, 200, and 300 μM, and daidzein 30 μM plus hesperetin 50, 100, and 150 μM) or their vehicle was cumulatively added to the organ bath. At the end of the experiment without washout, 1 mM of aminophylline was added to standardize the tissues relaxing maximally (100%).

### Statistical Analysis

The interaction of drug combination was evaluated as interaction index (*I*) < 1, = 1 or >1 represents synergism, zero-interaction or antagonism, respectively (Berenbaum, 1989; Wagner and Ulrich-Merzenich, 2009).

All values including drug combination in isobole are given as the mean ± SEM (*n*), *n* is the number of experiment. The difference between two values was determined by Student’s *t*-test. Differences with *P <* 0.05 were considered statistically significant.

## Results

### Sensitized Guinea-Pig Trachea

**FIGURE 1:**
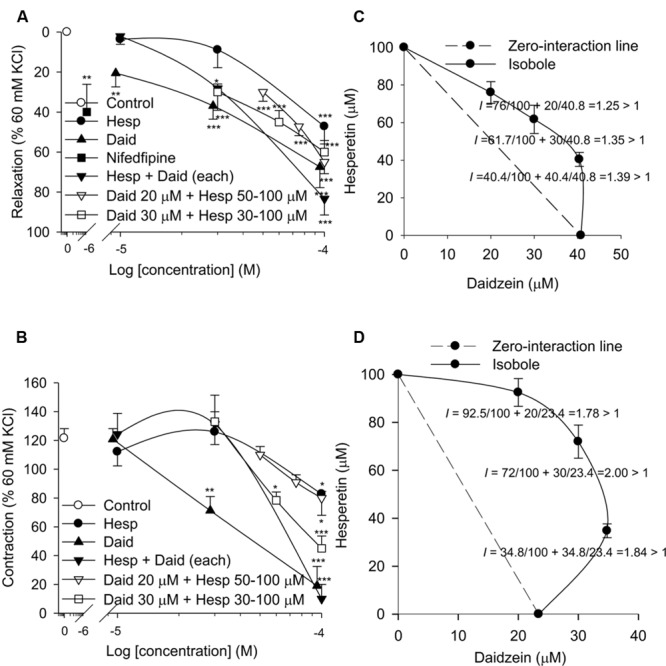
**Relaxing effects of daidzein (daid), hesperetin (hesp), and combination of both on baseline **(A,C)** and on OVA (100 μg/ml)-induced contraction **(B,D)** in isolated sensitized guinea-pig trachealis.** All data including combination of daidzein and hesperetin in isoboles **(C,D)** are the mean ± SEM. The number (*n*) of experiment was 6∼10. ^∗^*P* < 0.05, ^∗∗^*P* < 0.01, ^∗∗∗^*P* < 0.001 when compared to the control (vehicle). *I*: interaction index.

The KCl (60 mM)-induced contraction was 715 ± 189 mg (*n* = 11). Daidzein (10, 30, and 100 μM), hesperetin (100 μM), or combination (daidzein plus hesperetin 30 and 100 μM each, daidzein 20 μM plus hesperetin 50, 75, and 100 μM, and daidzein 30 μM plus hesperetin 30, 60, and 100 μM) significantly relaxed the baseline when compared to the control. Nifedipine (1 μM), a blocker of calcium channels, significantly relaxed the baseline about 40% of the KCl (60 mM)-induced contraction (**Figure 1A**). Isobole showed antagonism between daidzein and hesperetin on the baseline relaxant effect in the sensitized guinea-pig trachea (**Figure 1C**). Cumulative OVA (0.01, 0.1, 1, 10, and 100 μg/ml) concentration-dependently evoked contractions to the maximum of 589 ± 105 mg (*n* = 11), which was 121.5 ± 6.7% of the KCl (60 mM)-induced contraction. Daidzein (30 and 100 μM), hesperetin (100 μM), or combination (daidzein plus hesperetin 100 μM each, daidzein 20 μM plus hesperetin 100 μM, and daidzein 30 μM plus hesperetin 60 and 100 μM) significantly inhibited the contraction when compared to the control (**Figure 1B**). Nifedipine (1 μM) did not affect these contractions (data not shown). Isobole showed antagonism between daidzein and hesperetin on the OVA (100 μg/ml)-induced contraction in the sensitized guinea-pig trachea (**Figure 1D**).

### Non-sensitized Guinea-Pig Trachea

**FIGURE 2:**
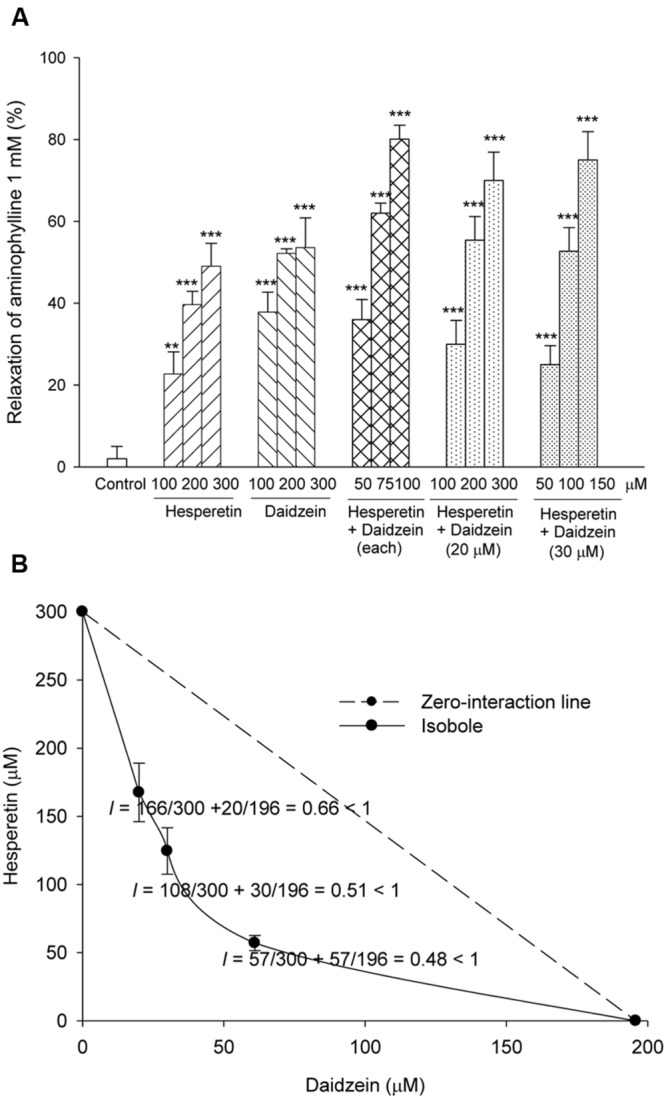
**Relaxing effects of daidzein, hesperetin, and combination of both on histamine (30 μM)-induced tonic contractions **(A,B)** in isolated non-sensitized guinea-pig trachealis.** All data including combination of daidzein and hesperetin in isobole **(B)** are the mean ± SEM. The number (*n*) of experiment was 6∼10. ^∗^*P* < 0.05, ^∗∗^*P* < 0.01, ^∗∗∗^*P* < 0.001 when compared to the control (vehicle). *I*: interaction index.

Histamine (30 μM) evoked a tension of 920 ± 50 mg (*n* = 16) in isolated non-sensitized guinea-pig trachea. Daidzein (100, 200, and 300 μM), hesperetin (100, 200, and 300 μM) or combination (daidzein plus hesperetin 50, 75, and 100 μM each, daidzein 20 μM plus hesperetin 100, 200, and 300 μM, and daidzein 30 μM plus hesperetin 50, 100, and 150 μM) significantly and concentration-dependently relaxed the histamine-evoked contractions (**Figure 2A**). Isobole showed synergism between daidzein and hesperetin in the tracheal relaxation (**Figure 2B**).

## Discussion

In isolated sensitized guinea-pig trachea, nifedipine (1 μM) significantly relaxed the baseline about 40%, but did not affect cumulative OVA (0.01∼100 μg/ml)-induced contractions, which may involve the release of tachykinins from sensory nerve ending (Bertrand et al., 1993), suggesting that the baseline tension is maintained in a part by a Ca^2+^ influx through L-type of voltage-dependent calcium channels from the extracellular fluid. The nifedipine-insensitive baseline tension was unrelated to prostaglandins as indomethacin (3 μM) present throughout the experiment. However, daidzein (10, 30 and 100 μM), hesperetin (100 μM), and combination (daidzein plus hesperetin 30 and 100 μM each, daidzein 20 μM plus hesperetin 50, 75, and 100 μM, and daidzein 30 μM plus hesperetin 30, 60, and 100 μM) significantly relaxed the baseline, suggesting that the nifedipine-insensitive baseline tension is related to PDE3 and PDE4 activities, but other isozymes may be involved (Silver et al., 1988). The inhibitory effects of daidzein (30 and 100 μM) on the OVA (100 μg/ml)-induced maximal contraction were obviously greater than those of hesperetin (30 and 100 μM), although their IC_50_ values (28.6 and 28.2 μM, respectively) on partially purified PDE3 and PDE4 are similar (Ko et al., 2004). These results suggest that the PDE3 activity may decrease or/and PDE4 activity increase after sensitization. This results in that daidzein is more potent than hesperetin at the same concentration. Both isoboles showed antagonism between daidzein and hesperetin. Our present results revealed an antagonism between hesperetin and daidzein are consistent with the previous report (Clayton et al., 2004).

In non-sensitized guinea-pig trachea, the isobole showed relaxing synergism between daidzein and hesperetin on histamine-induced tonic contraction. Consistently, the bronchodilatory and anti-inflammatory effects of dual PDE3/4 inhibitors are more obvious when compared with those of either isozyme inhibition (Abbott-Banner and Page, 2014). These results point to differing effects in patients with or without allergic asthma. From TCM such examples are known, e.g., in clinic, Sun-Su-In, a preparation containing *Panax ginseng, Lithospermi* radix, *Glycyrrhizae* radix, kudzu, Chen-Pi, etc., is mainly used for relieving infection of upper respiratory tract, bronchitis, pneumonia, and emphysema in children or elderly patients without asthmatic history (Zuong, 1995).

## Conclusion

These findings in sensitized and non-sensitized guinea-pig tracheas suggest that the combination of kudzu and Chen-Pi for relieving colds, fever, bronchitis and cough is effective in patients without, but might show little effect in patients with allergic asthma.

## Author Contributions

C-HS and W-CK conceived and designed the study. T-YC performed the experiments and analyzed the data. C-HS and W-CK wrote the manuscript. All the authors read and aproved the final manuscript.

## Conflict of Interest Statement

The authors declare that the research was conducted in the absence of any commercial or financial relationships that could be construed as a potential conflict of interest.

## Abbreviations

cAMP: adenosine 3′,5′ cyclic monophosphate
cGMP: guanosine 3′,5′ cyclic monophosphate
DMSO: dimethylsulfoxide
OVA: ovalbumin
PDE: phosphodiesterase
TCM: traditional Chinese medicine
